# The King’s Daughters’ Hospital

**Published:** 1888-10

**Authors:** 


### "THE KING'S DAUGHTERS' HOSPITAL."

The city of Atlanta is to be congratulated upon the inauguration of this excellent and charitable institution, the doors of which were thrown open to those requiring its kindly ministrations on the 1st of August.

Originating under the auspices of the Episcopal Church, it is yet pleasing to note that this noble charity is non-sectarian in character, embracing as it does all Christian creeds, and recognizing no dominant sect, either in the range of its charities or in the selection of its Board of Trustees and corps of medical managers. The hospital has been well named, after the charitable order of "The King's Daughters," an organization now well known for their devoted works of mercy and love amongst the sick and suffering poor. To the noble women of this city too much praise cannot be accorded for their untiring interest and substantial aid in forwarding this most deserving institution, the work which they have already accomplished being an earnest of the undoubted success of the hospital. It is but fitting and just that, fulfilling the time-honored maxim, "Honor to whom honor is due," we state that to Dr. Augustus J. Woodward, of this city, more than to any other individual, is due the credit of the inception and successful inauguration of this much-needed institution. This gentleman, well known for his untiring and charitable work amongst the sick-poor of St. Luke's parish, has labored with unflagging zeal and against all discouragement to accomplish the object of his ambition, namely, the establishment of a hospital for the sick-poor, where proper attention and treatment for all diseases should be afforded. Enlisting first the sympathy and aid of the clergy and Bishop of his own communion, he has seen his efforts develop into a movement, which, broadening in its sweep, has cleared the bounds of church and creed, enlisting the aid and appealing to the sympathy of "those who love their fellowmen."

The Board of Trustees is composed of the following gentlemen:

President, Rt. Rev. John W. Beckwith; E. P. Chamberlin, W. M. Dixon, E. C. Peters, J. M. Green, S. M. Inman, John A. Fitten, Paul Romare, H. W. Grady, Z. D. Harrison, Rev. E. H. Barnett, Rev. Byron Holly, Rev. Augustine Prentice, Dr. A. J. Woodward, Dr. J. P. Logan, Dr. Geo. H. Noble, Secretary; Oliver Cranston, Treasurer.

EXECUTIVE COMMITTEE—John A. Fitten, E. C. Peters, A. J. Woodward.

MEDICAL STAFF—General Medicine—Dr. J. C. Olmsted; General Surgery—Dr. W. F. Westmoreland, Jr.; Diseases of Women—Dr. Geo. H. Noble; Obstetrics and Children—Dr. Augustus J. Woodward; Diseases of Eye and Ear—Dr. J. M. Crawford; Diseases Throat and Nose—Dr. F. O. Stockton.

BOARD OF CONSULTANTS—Drs. A. W. Calhoun, W. F. Westmoreland, Sr., W. S. Armstrong, J. P. Logan, W. A. Love, H. Bak, W. P. Nicolson, R. B. Ridley, J. F. Todd, K. C. Divine, J. J. Knott, T. D. Longino, Virgil O. Hardon, W. R. Waring, J. F. Alexander, J. B. Baird and A. G. Hobbs.

"O."

### ITEMS.

WE regret to have to announce the death of Mrs. Crawford W. Long, the wife of the distinguished discoverer of Anæsthesia (whose portrait graces the title-page of this journal), which occurred in a railroad accident near San Antonio, Texas, September 22d, 1888.

THE thirty-first annual course of lectures in the Atlanta Medical College will open October 3d, 1888. The opening address will be made by Dr. Wm. Abram Love.

NOTWITHSTANDING the presence of a large number of refugees in Atlanta from Jacksonville and other infected places, the health of the city was never better. Not a single case of yellow fever has occurred in the city or its vicinity; in fact, not a case is known to exist in the State of Georgia.

THE annual session of the Southern Medical College will commence October 2d, 1888. Dr. J. McF. Gaston will make the opening address.

A CATCHER KILLED BY A BASE-BALL.—Henry Zickemeyer, of Tiffin, Ohio, catcher of the Bloomville Base-ball Club, was instantly killed, on August 30th, during the progress of a game. He was struck over the heart by a foul tip and dropped dead after returning the ball to the pitcher.—*Ex.*

THE SOUTHERN SURGICAL AND GYNECOLOGICAL ASSOCIATION.—The meeting of the Association will not be held in Birmingham on the 11th, 12th and 13th of September as announced, but has been postponed till the first Tuesday in December, owing to quarantine against yellow fever.

WE note that *The Canada Medical and Surgical Journal* has changed its name to *The Montreal Medical Journal;* it has been enlarged in size from sixty-four to eighty pages; its subscription rate has been reduced to $2.00 per annum, and altogether, under its new name, it is a most promising journal, and we wish it all possible success.—*Ex.*

J. B. LIPPINCOTT COMPANY take pleasure in announcing that a new edition of the *United States Dispensatory* is now being bound, and will be ready in a few days. The revision has been thorough, and not merely the addition of a supplement. More than one-third of the book, or nearly eight hundred pages, is entirely new matter, while the whole work has been most carefully re-written. The National Formulary has been incorporated.

AN AWFUL BLUNDER.—Druggist (in alarm)—"James, run to Mrs. Smith's at once; I've made an awful mistake!" James (seizing his hat)—"Morphine—quinine—arsenic—poison—" Druggist—"No, no; she sent for ten cents' worth of one-cent stamps, and I sent her ten *twos!*"—*The Sun.*

THE NEW YORK POLYCLINIC.—The Seventh Annual Announcement shows an attendance of 337 practitioners for the session of 1887–'88. The only changes in the faculty are the appointments of Dr. Charles Stedman Bull as professor of ophthalmology, and Dr. Henry N. Heinman as professor of general medicine. The session of 1888–'89 will open on Sept. 24th.

A WEST END thief, says the *Boston Journal*, who was arrested recently for drunkenness, said that it was a downright shame. "For," said he, "I am a thief, and a good one, and it will hurt my reputation among my pals to be arrested for simply being drunk. If it was for robbery, or something like that, I wouldn't care, but I don't want to be arrested for drunkenness."—*Ex.*

FEARFUL MISTAKE.—Two young men, Horace Bishop and Charles Applebee, aged sixteen, of Branford, Conn., stopped at a drug-store, in passing through the town, and called for soda-water and brandy, which was given them. They shortly after returned in a very sick condition. An investigation revealed the fact that tincture of aconite had been mixed with the soda-water instead of brandy. Both died from the effects in about half an hour. The clerk was arrested.—*Ex.*

GROWING A TREE IN HIS WINDPIPE.—Alvey Clabaugh, a youth of about twelve years, residing with his father in Frederick, Md., about four years ago swallowed a persimmon seed, which was supposed to have lodged in his throat, and which at times caused him considerable inconvenience. Several days ago it became quite painful, and a doctor was called in, who stated that the seed was sprouting where it had lodged in his windpipe. —*Ex.*

FRENCH JUSTICE IN A CASE OF MISTAKE IN PRESCRIPTION-WRITING.—A French medical man recently prescribed one gramme of sulphate of atropine instead of a centigramme, the error resulting in the death of his unfortunate patient. The doctor and the chemist who dispensed the prescription have been found guilty of homicide through imprudence, and the former was condemned to pay 600 francs as compensation, and the latter to five days' imprisonment and the payment of 400 francs.

A TERRIBLE DIAGNOSIS.—A certain fashionable Indianapolis lady visited an old friend, a Cincinnati doctor, accompanied by her daughter. "There is something the matter with Jenny's voice," remarked the anxious mother. After a careful examination the doctor solemnly said, "Madam, your daughter has a mezzo-soprano." "What!" exclaimed the mother bursting into tears. "Then she is incurable; that's what carried the Emperor William off, according to Mackenzie." Fee paid and doctor smiled.—*Lancet & Clinic.*

THE DOCTORS AND THE YELLOW FEVER EPIDEMIC.—"The whole country has observed with admiration the heroic conduct of the physicians who are battling with the yellow fever in the stricken city of Jacksonville. They have not only remained at the post of duty in the presence of danger, rendering services freely to the needy, but they have striven to surpass each other in deeds of devotion and self-sacrifice. Several of them have fallen victims to the plague during the past two months, and one of the most grievous incidents reported from Jacksonville is the death, on Monday last, of Dr. W. L. Baldwin, who caught the disease from one of his patients in the hospital. Not a few doctors practicing in different parts of the country have gone to Jacksonville to render relief to the distressed people, and hundreds of others have nobly offered to follow their example. Truly such physicians are worthy of honor and gratitude from mankind."—*New York Sun.*

THE ONLY REMEDY.—Doctor, to a *malade imaginaire:* "There is but one remedy that can save you." Patient : "And what is that ?" Doctor: Get your daughter married. "You are then a mother-in-law, and they are notoriously very tenacious of life."—*Fliegende Blätter.*

BOSTON papers tell of "the singular death, at Danvers, of Miss Emma Felch. She was taken ill some months ago, and, from the fact that her mother died of cancer, she became possessed with the idea that her sickness was from the same cause. Her physicians could find no indication of cancer, but she asserted that she had one, and located it. She refused food, saying it distressed her. At her desire, after she died, an autopsy was held, and no cancer could be found. It was decided that her disease was purely sympathetic."

AN UNPREMEDITATED CÆSAREAN SECTION.—The town of Titusville, Pa., has been very much excited over an unpremeditated Cæsarean section which was recently performed there by Dr. Varian. It seems that a young woman who was far advanced in pregnancy succeeded in imposing upon the credulity of her physicians to such an extent that she was thought to be suffering with an ovarian tumor. Pregnancy was suspected, but absence of the fœtal heart-sounds, taken in connection with the young woman's positive declarations that she could not be pregnant, led to the mistake in diagnosis. In an operation undertaken for the removal of the supposed tumor, the uterus was opened and a living child, weighing about eight pounds, extracted. Dr. Varian's own account of the case will be published soon.—*Medical and Surgical Reporter.*

